# Continuous nursing for infants with congenital talipes equinovarus undergoing Ponseti therapy and telehealth education for their parents via WeChat: a single center retrospective study

**DOI:** 10.3389/fpubh.2024.1399616

**Published:** 2024-07-22

**Authors:** Xiangxuan Wang, Kainan Lin, Jinrun Lin, Wenchen Xu, Hui Chen

**Affiliations:** ^1^Department of Pediatric Orthopedics, Fujian Maternity and Child Health Hospital College of Clinical Medicine for Obstetrics & Gynecology and Pediatrics, Fujian Medical University, Fuzhou, China; ^2^Department of Pediatric Orthopedics, Fujian Children's Hospital (Fujian Branch of Shanghai Children’ Medical Center), Fuzhou, China

**Keywords:** continuous care, telehealth education, WeChat group, complications, care burden, congenital talipes equinovarus, Ponseti treatment, parents

## Abstract

**Aims:**

This study aimed to evaluate the impact of continuous nursing and telehealth education via WeChat in infants with congenital talipes equinovarus (CTEV) undergoing Ponseti therapy on reducing complications, care burden, and improving the quality of life for parents.

**Methods:**

This is a single-center retrospective study. From July 2021 to December 2022, 44 CTEV children who undergoing Ponseti treatment in our hospital who received continuous nursing and telehealth education via WeChat (experimental group). In addition, during January 2020 to June 2021, 44 children with CTEV treated with Ponseti in our hospital who received routine nursing and traditional health education were selected as the control group. The incidence of complications, parental care burden and parental quality of life were compared between the two groups.

**Results:**

There was no significant difference in the demographic characteristics of patients and parents between the two groups, and the groups were comparable (*p* > 0.05). The incidence of complications including plaster loosens, plaster falling off, pressure ulcer was significantly lower in the intervention group compared to the control group (*p* < 0.05). Parents in the intervention group experienced significantly lower care burdens compared to those in the control group (*p* < 0.05). The quality of life of parents in the intervention group was significantly higher than that for the control group (*p* < 0.05). There were significant differences in the incidence of complications, the care burden of parents and the quality of life of parents between the two groups.

**Conclusion:**

This study found that continuous nursing and telehealth education via WeChat group during Ponsetis treatment of children with CTEV can effectively reduce complications, reduce the care burden of parents and improve the quality of life of parents. This method is simple and convenient, especially worthy of application and promotion in medically underdeveloped areas.

## Introduction

Congenital talipes equinovarus (CTEV) is a frequent birth anomaly marked by equinus, hindfoot varus, adductus of the forefoot, and cavus deformities (1). The frequency of clubfoot is approximately 1 in 1,000 live births (2, 3), with rates varying from 0.51 per 1,000 in the Chinese population (4). In this century, the initial treatment standard for CTEV worldwide is to use the Ponseti method for manipulation and serial casting (5). Previous investigations have shown that 50% of children treated with the Ponseti technique relapsed and required further plaster or surgical treatment (2).

Parents of children with CTEV often experience increased anxiety and stress, similar to the challenges faced by parents of disabled children, potentially leading to family dysfunction (6, 7). Our conversations with parents of children with CTEV showed that many parents felt stress, anxiety and worry about home care during Ponseti treatment. They worried that complications would occur due to their negligence or improper observation during the treatment, which would affect the therapeutic effect of the affected foot and have a significant impact on the long-term efficacy.

Parents must offer adequate home care for children with CTEV while undergoing Ponseti therapy. Due to some problems such as living far away from the hospital, such as when parents finding some problems in home care, lack effective means of communication with term health professionals (including specialist doctors and nurses). We often find that some parents spend hours consulting doctors in hospitals because of a small problem. Is it possible to solve this problem through some mobile social media?

Recent years, WeChat has firmly established itself in the daily lives of Chinese people as the most widely used mobile social media program in China, boasting over 1.3 billion registered users. WeChat facilitates various daily tasks including instant messaging through text, images, and voice (8, 9). WeChat has been used to perform telehealth and store a large volume of patient-generated health data (8, 10). However, while utilizing WeChat as a health education and remote care tool, it is important to examine the legal and regulatory challenges around data ownership and use. Patients, clinicians, and researchers all have challenges in protecting their privacy and security (11). Although China has made appropriate efforts to protect personal information security (12), WeChat’s privacy and security management systems still have certain flaws.

A study shows that compared with traditional health education technology, WeChat-based health education is more successful (13). There is no report on the application effect of continuous nursing and distance education via WeChat group on children with CTEV during Ponseti treatment. We hypothesized that WeChat group-based continuous care and remote health education can reduce complications in children with CTEV during Ponseti treatment and reduce the care burden of parents and improve the quality of life of parents. The purpose of this study was to evaluate the clinical efficacy of this method.

## Methods

The present study was approved and supervised by the Ethics Committee of Fujian Maternal and Child Health Hospital, and was conducted in accordance with the ethical standards set out in the 1964 Declaration of Helsinki. Additionally, all the parents of the children included in the study agreed that the children and themselves participated in the study and signed a written informed consent form.

### Sample size calculation

Based on the pre-experiment data and assuming an alpha value of 0.05 with a power of 0.90, the needed number of participants was determined to be 40 in each group. Assuming a 10% missing rate, the total sample size was set at 88 (44 each group).

### Research data acquisition

This study is a single-center retrospective study and was conducted at the Children’s Orthopaedic Centre of a provincial children’s hospital in southeastern China.

From January 2020 to June 2021, clinical data and parent-related scale scoring results of 44 patients with CTEV who underwent treatment with the Ponseti method were retrospectively collected via the electronic medical record system. Routine care was administered to the children during the treatment period, and parents were provided with health education materials, this group as a control group.

From July 2021 to December 2022, the clinical data and parent-related scale scoring results of 44 patients with CTEV who treated using the Ponseti method were retrospectively collected via the electronic medical record system. Throughout the treatment period, parents of the children in this cohort received ongoing care and remote guidance from specialized medical professionals through the use of WeChat group, this group as the experimental group.

### Inclusion and exclusion criteria

The following inclusion criteria were used: (1) infants with CTEV whose diagnosis was clearly treated at our hospital; (2) those who met the indications for treatment by the Ponseti method; (3) parents of children who were able to use the Internet and WeChat easily; and (4) complete clinical data. The following exclusion criteria were used: (1) patients who were not treated for the entire course of treatment at our hospital; (2) patients with other congenital diseases in combination; and (3) patients whose family members refused to participate in the study.

A comparative analysis of the data from the two groups was conducted. All patients were diagnosed with CTEV at birth and received Ponseti method treatment from two paediatric orthopaedic specialists at one month of age. The researcher screened eligible parents for study and collected relevant data.

### Grouping based on different interventions

#### Routine care and traditional health education (control group)

From January 2020 to June 2021, we offered routine care for children with CTEV treated with Ponsetis at our facility, and we used a health education handbook to provide parents customary health information.

Implementation of routine care: Specialist medical staff explained the pathogenesis, treatment, and precautions of CTEV to the parents of the children. They closely monitored the children’s conditions and promptly provided symptomatic treatment upon detecting abnormalities. Additionally, they instructed on manoeuvres, plaster fixation, body position, and provided parents with precautions and health education manuals. Parents were informed to promptly return to the hospital for follow-up in case of any condition.

#### WeChat group based continuous nursing and telehealth education (experimental group)

From January 2020 to June 2021, we are based on routine child care during the home period with the support of a WeChat group for continuity of care, as well as using a WeChat group to guide the patients’ parents. At the start of Ponseti treatment, healthcare personnel encouraged parents to join the WeChat group and trained them how to use it correctly and skillfully. It is separated into two major sections: home care education and Q&A. The home nursing evaluation includes knowledge and precautions for plaster care throughout Ponzi therapy, as well as the assessment and treatment of skin injuries. In the Q&A module, a healthcare worker is on duty every day to answer questions for parents online at set hours.

The CTEV-related WeChat group consists of a nurse from the speciality who is responsible for the operation of the group, and a specialist doctor who manages consultations with the children treated in the outpatient clinic and provides dynamic guidance to the families. The group regularly pushes out small videos to guide parents on home care, such as the precautions to be taken when immobilising a child in a plaster cast.

### Research tool

All parents were required to complete the Family Caregiver Task Inventory (FCTI), the Zarit Burden Interview (ZBI) and World Health Organization Quality of Life-BREF (WHOQOLBREF) scale before starting Ponseti treatment (pre-treatment), and completed the FCTI, ZBI, WHOQOLBREF scales at the end of the cast fixation (plaster removal). All clinical and family data are shown in Table 1.

**Table 1:** Demographic characteristics of patients and their parents in two groups.

	Experimental group (*n* = 44)	Control group (*n* = 44)	*p* value
**Age of patients (days)**	35.3 ± 2.7	33.4 ± 1.9	0.627
**Weight (kg)**	4.2 ± 2.2	4.3 ± 1.7	0.553
**Sex**			0.863
Female	17	19	
Male	27	25	
**Sides**			0.721
Left	8	11	
Right	19	17	
Bilateral	17	16	
**Age of parents (years)**			0.762
≤25	6	8	
26–30	18	19	
31–35	11	9	
36–40	7	5	
≥40	2	3	
**Parents’ education level**			0.574
Under high school	13	11	
High school	18	21	
Junior college	6	6	
Bachelor degree or above	7	6	
**Place of abode**			0.772
Rural area	32	28	
City	12	16	

#### Family caregiver task inventory

In this study, the FCTI scale in Chinese, utilized was a modification of the original FCTI scale developed by Clark et al. (14), as revised by Lee et al. (15). The scale comprises 25 items categorized into 5 dimensions: managing care responsibilities, offering help and support, addressing personal emotions, evaluating available resources, and adjusting personal and care-related obligations. Each item is rated on a 3-point Likert scale: 0 indicates not challenging, 1 indicates somewhat challenging, and 2 indicates highly challenging. The overall score ranges from 0 to 50, with higher scores indicating greater caregiving difficulties and decreased caregiving capabilities.

#### Zarit burden interview

The ZBI scale, designed by Zait et al. (16), was translated into Chinese in 2006 by Wang and Hou (17). This assessment tool comprises 22 items, measuring two key aspects: individual burden and role burden. Evaluate caregivers’ burden overall with Item 22. Respondents rate each item using a 5-point Likert scale: “no,” “occasionally,” “sometimes,” “often,” and “always” correspond to 0, 1, 2, 3, and 4 points, respectively. A higher total score indicates a greater care burden, with a maximum score of 88. Scores below 19 suggest a minimal burden, while 20 to 39 represent a moderate burden, 40 to 59 indicate a significant burden, and over 60 signify a severe burden.

#### World health organization quality of life-BREF

The WHOQOL-BREF scale, derived from the WHOQOL-100 scale (18), consists of 26 items that assess various aspects of quality of life: physical well-being, psychological well-being, social relationships, and environment. Items 1 and 2 address different topics and their combined scores serve as an overall quality of life indicator. Each item is rated on a 1–5 scale, with items 3, 4, and 26 being negatively scored, reversing the scale from 5–1. Higher scores indicate better quality of life.

### Statistical analysis

Continuous variable were reported as the mean range and standard deviation. A normal distribution test was conducted on all continuous variable (Shapiro–Wilk test), which showed adherence to a normal distribution. Comparison of clinical parameters between the two groups was carried out using the independent samples t-test, Levene ‘s test was used to test the homogeneity of variance, and the variance between the two groups was consistent. Variables were categorized using either the χ^2^ test or Fisher’s test. Statistically significant differences were defined as *p*-values <0.05. SPSS (Windows version 22.0 IBM Co., Armonk, NY, United States) was used for all statistical analyses.

## Results

### Demographic characteristic

There was no significant difference in demographic characteristics between the two groups of patients and their parents (Table 1). According to the findings, 68.1% of the children live in rural areas, while 72.7% of the parents of the patients had a high school diploma or lower education level.

### Comparison of FCTI, ZBI score and complications between groups

Prior to treatment, there was no significant difference in FCTI and ZBI scores between the two groups. After removing the plaster, the FCTI score of the experimental group was significantly lower than that of the control group (*p* = 0.021) and the FCTI score of the experimental group was significantly lower than that before treatment, while the FCTI score of the control group was not significantly reduced (Table 2).

**Table 2:** Comparison of the complication of patients, FCTI and ZBI score of parents between the two groups.

	Experimental group	Control group	*p* value
Pre-treatment			
Score of FCTI	42.1 ± 5.0	42.2 ± 4.9	0.453
Score of ZBI	61.1 ± 9.5	60.0 ± 10.9	0.672
Plaster removal			
Score of FCTI	32.8 ± 5.6	36.2 ± 4.9	0.021*
Score of ZBI	26.8 ± 8.3	43.8 ± 13.7	0.019*
Complications	2 (2 feet)	5 (9 feet)	0.033*
Plaster loosens	2 (2 feet)	3 (5 feet)	
Plaster falling off	0	1 (2 feet)	
Pressure ulcer	0	1 (2 feet)	

* The difference between two groups was statistically significant (*p* < 0.05). FCTI, Family Caregiver Task Inventory; ZBI, Zarit Burden Interview.

After removing the plaster, the ZBI score of the experimental group was significantly lower than that of the control group (*p* = 0.019). Compared with pre-treatment, the ZBI score of the experimental group decreased significantly, while the ZBI score of the control group did not decrease significantly (Table 2).

In terms of complications, The difference between the two groups was statistically significant (*p* = 0.033) (Table 2). Two patients (2 feet) in the experimental group developed loose casts during Ponseti treatment, and the parents sent the pictures to the term health professionals through WeChat, and the doctor suggested returning to the hospital in time to replace the plaster, and no complications occurred. Of the 40 patients in the control group, there were 5 cases (9 feet) with complications, of which 3 cases (5 feet) had loose casts, due to the parents failed to identify in a timely manner, until the replacement of plaster as originally planned, local skin had already been broken, but fortunately, after a simple disinfection and change of medication and continued to perform plaster fixation, the incision eventually healed without affecting the progress of treatment. In one case (2 feet), the plaster came off, and in one case (2 feet), a pressure sore on the heel skin (stage II pressure sore) developed, and we had to stop the plaster fixation and prioritise the treatment of the wound. The treatment was eventually continued after two weeks of dressing changes and wound healing.

### Comparison of WHOQOL-BREF score between groups

Scores of WHOQOL-BREF within the physical, psychological, social, and environmental realms were notably elevated in the WeChat group-based continuous nursing and telehealth education groups in comparison to the routine care and traditional health education groups (*p* < 0.05). Moreover, the cumulative quality of life rating in the experimental cohort surpassed that of the control group with statistical significance (*p* < 0.05) (Table 3).

**Table 3:** Comparison of the WHOQOL-BREF score of parents between the two groups.

	Experimental group	Control group	*p* value
Physiological fields	12.7 ± 1.5	10.3 ± 1.4	0.027*
Psychological fields	13.9 ± 1.9	9.8 ± 1.2	0.030*
Social fields	14.5 ± 2.4	10.5 ± 1.4	0.031*
Environmental fields	12.9 ± 1.8	10.2 ± 1.3	0.023*
Total quality of life scores	62.6 ± 6.7	48.4 ± 7.1	0.024*

*The difference between two groups was statistically significant (*p*<0.05). WHOQOL-BREF, World Health Organization Quality of Life-BREF.

## Discussion

Congenital talipes equinovarus is the most common developmental malformation in children, which does not belong to embryonic malformation (19, 20). In the 4–6 month of pregnancy, it is common for talipes equinovarus to develop. According to related research (19–21) CTEV babies born in underdeveloped nations account for around 80% of the global total. If clubfoot is not properly treated, it may lead to long-term dysfunction and decreased quality of life (22). Ponseti method is a method developed by Dr. Ignacio Ponseti for the treatment of clubfoot (2). This method has the characteristics of simple operation, significant curative effect and low cost. It has become the first choice for the treatment of CTEV. However, the Ponseti method has a long treatment time, and most of them require parental home care. Improper care can lead to complications such as gypsum shedding and skin pressure sores, which can affect the final treatment effect in severe cases.

It is common knowledge that the sickness and hospitalization of children can lead to a crisis within the family and cause anxiety for parents (23, 24). The anxiety experienced by parents is often a result of a lack of understanding and information regarding the illness and medical care (25–27). Our study show that continuous nursing and telehealth education via WeChat group is effective in the parents of children with CTEV during the treatment of Ponseti therapy, and that tele-education improves parental caregiving, reduces the nursing load, lowers the rate of loss of visits, and reduces complications. We used FCTI, STAI and WHOQOL-BREF rating scales to assess the parents of children at different stages. Comparing the experimental group with the control group, the results showed that the experimental group had a statistically significant reduction in all scores during treatment compared to before treatment. There was a decrease in the scores of the control group, but the difference was not significant. Interestingly, there was a significant difference between the two groups in terms of complications, only 2 feet in the test group had loose casts, and the parents communicated with the doctors through WeChat in a timely and effective manner, and the replacement of the casts had an impact on the patients. Complications occurred in 5 patients (9 feet) in the control group, 3 patients (5 feet) have been loosened during the period of home, the family failed to deal with in time, to be returned to the hospital for treatment according to the booking time, the local skin has been broken, these 5 patients by disinfection and dressing change treatment did not lead to serious consequences, 1 case (2 feet) of patients with pressure sores on the heel during the period of gypsum immobilisation, We had to give priority to the trauma and suspend the plaster treatment.

Nowadays, many parents still lack adequate knowledge about caring for their children at home. The concentration of advanced medical resources in urban centers has led to disparities in healthcare access, with rural areas, particularly in China, lacking in basic medical services. The popularization of children ‘s orthopedic knowledge is limited in rural areas. In this study, 68.1% of children reside in rural regions, and their parents must go to large city hospitals to address their children’s health issues. They only need a few minutes of outpatient consultation services, and need a greater economic burden and time cost. This often increases the care load on parents, which is especially essential for patients with CTEV treated with Ponseti, because they must pay close attention to the patient’s condition in addition to visiting the hospital every week in order to discover and treat the problem.

In this study, 72.7% of the patients’ parents had a high school diploma or less. Health literacy is limited, and they simply cannot comprehend the facts by reading a basic leaflet. They are concerned that a lack of preparation may result in problems, poor results, and a high level of anxiety. The study found that compared with the general population, the parents of children with CTEV have more stress and worry (28), and the proportion of depressive symptoms is higher (29). As a result, it is critical for these parents to investigate a more effective guiding strategy for CTEV children during Ponseti therapy.

With the advancement of mobile information technology in recent years, smartphones have become prevalent globally, resulting in the increasing utilization of telemedicine services (30). Various social media platforms are extensively utilized in managing health and educating individuals about chronic illnesses like diabetes (31). The purpose of this is to improve clinical outcomes and reduce the pressure on patients and their families (10). According to existing research findings, WeChat surpasses conventional approaches as a health education tool in managing diseases (32, 33). It has been proven to lower time and financial expenditures, enhance treatment compliance, decrease patient complications, boost post-treatment monitoring rates, and ameliorate patient well-being (9). This study we hypothesized that WeChat group-based continuous care and remote health education can reduce complications in children with CTEV during Ponseti treatment and reduce the care burden of parents and improve the quality of life of parents, final results confirm our hypothesis.

There are some limitations in this study. Firstly, this is a retrospective study, may be some bias in case selection, and the statistical efficacy of retrospective study is lower than that of prospective study, it is necessary to conduct prospective studies in the future to further verify the findings of this study. Secondly, this study is a single-center study, which limits the wide implementation and promotion of the research results. Fortunately, we have begun to promote it in some collaborative hospitals. In the future, we will to carry out multi-center and multi-disciplinary collaborative research to confirm the feasibility of this study. Thirdly, this study does not use randomization, which may lead to uncontrolled variables affecting the results. In our study, patients were strictly included according to the inclusion and exclusion criteria. In the future, we intend to carry out a multi-center prospective randomized controlled study to confirm the reliability of the results. Finally, although the use of smartphones and WeChat applications are relatively popular, there are still parents without Internet or smartphones who are unable to participate in this study, and caregivers of children may change during treatment, resulting in information lag.

## Conclusion

Continuous nursing and telehealth education via WeChat group can effectively reduce complications in infants with CTEV undergoing Ponseti therapy, reduce the care burden of parents and improve the quality of life of parents. Results of this study can lay a foundation for the development of multi-center prospective controlled studies in the future. This measure not just affordable and convenient, but also interactive, especially worthy of application and promotion in medically underdeveloped areas.

## Data availability statement

The raw data supporting the conclusions of this article will be made available by the authors, without undue reservation.

## Ethics statement

The studies involving humans were approved by Ethics Committee of Fujian Children’s Hospital. The studies were conducted in accordance with the local legislation and institutional requirements. Written informed consent for participation in this study was provided by the participants’ legal guardians/next of kin. Written informed consent was obtained from the individual(s), and minor(s)’ legal guardian/next of kin, for the publication of any potentially identifiable images or data included in this article.

## Author contributions

XW: Writing – review & editing, Investigation, Conceptualization, Writing – original draft, Funding acquisition, Formal analysis, Data curation. KL: Supervision, Software, Investigation, Writing – original draft, Data curation. JL: Resources, Project administration, Writing – original draft, Investigation. WX: Investigation, Writing – original draft, Supervision, Software. HC: Writing – review & editing, Visualization, Validation, Supervision.
